# Association between uric acid to high-density lipoprotein cholesterol ratio and chronic kidney disease in Chinese patients with type 2 diabetes mellitus: a cross-sectional study

**DOI:** 10.3389/fnut.2025.1582495

**Published:** 2025-04-14

**Authors:** Xiangyu Chen, Jie Zhang, Feng Lu, Ruying Hu, Xiaofu Du, Chunxiao Xu, Mingbin Liang, Lijin Chen, Weiyuan Yao, Zhimin Ma, Jieming Zhong, Meng Wang

**Affiliations:** Department of Non-Communicable Disease Control and Prevention, Zhejiang Provincial Center for Disease Control and Prevention, Hangzhou, China

**Keywords:** type 2 diabetes mellitus, high-density lipoprotein cholesterol, uric acid, chronic kidney disease, uric acid to high-density lipoprotein cholesterol ratio

## Abstract

**Objectives:**

To examine the association between uric acid (UA) to high-density lipoprotein cholesterol (HDL-C) ratio (UHR) and chronic kidney disease (CKD) in type 2 diabetes mellitus (T2DM) patients in China.

**Methods:**

The investigation stems from a survey conducted in the eastern Chinese province of Zhejiang, spanning from March to November 2018. A multivariable logistic regression model was employed to assess the relationship between UHR and CKD, while restricted cubic spline (RCS) analysis was used to evaluate the dose–response relationship. Receiver operating characteristic (ROC) curve analysis was performed to determine the optimal UHR cut-off value and assess its diagnostic performance for CKD. Model performance was further evaluated using net reclassification improvement (NRI) and integrated discrimination improvement (IDI) metrics. Sensitivity analyses, including propensity score matching (PSM) and k-means clustering, were conducted to enhance the robustness of the findings. Subgroup analyses were performed across various demographic and clinical categories to examine the consistency of the UHR-CKD association.

**Results:**

This cross-sectional study included 1,756 Chinese patients with T2DM, among whom 485 (27.62%) were identified with CKD. Multivariable logistic regression analysis revealed a significant positive association between UHR and CKD. Per standard deviation (SD) increase in UHR was associated with a 40% higher odds of CKD (OR = 1.40, 95% CI: 1.23–1.60) after adjusting for potential covariates. When analyzed categorically, participants in the highest UHR tertile (T3) had 1.82-fold higher odds of CKD compared to the lowest tertile (T1) (95% CI: 1.32–2.50). RCS analysis demonstrated a consistent linear dose–response relationship between UHR and CKD across all models (all p for nonlinearity >0.05). ROC curve analysis identified an optimal UHR cut-off value of 12.28 for CKD prediction, with an area under the curve (AUC) of 0.710 (95% CI: 0.683–0.737) in the fully adjusted model. Subgroup analyses confirmed the robustness of the UHR-CKD association across most demographic and clinical variables, except for younger age groups (18–44 and 45–59 years) and smokers. Notably, BMI significantly modified the UHR-CKD relationship, with a nonlinear association observed in individuals with lower BMI (<24 kg/m^2^) and a linear association in those with higher BMI (≥24 kg/m^2^).

**Conclusion:**

This study demonstrates a significant dose–response relationship between the UHR and CKD in Chinese patients with T2DM, highlighting UHR as a promising biomarker for CKD risk assessment. The identified UHR cut-off of 12.28 offers a practical threshold for early renal monitoring and targeted interventions. Future research should explore UHR-targeted therapies and its integration into personalized risk stratification models to improve CKD management in T2DM.

## 1 Introduction

Chronic kidney disease (CKD) represents an escalating public health concern worldwide, particularly in the context of type 2 diabetes mellitus (T2DM) (1). As a leading microvascular complication associated with diabetes, CKD plays a substantial role in elevating morbidity and mortality in those impacted (2). Current estimates indicate that CKD impacts over 10% of individuals worldwide, with over 850 million individuals worldwide living with this condition, and its prevalence is projected to rise further in the coming decades due to the escalating burden of diabetes, hypertension, and obesity (3, 4). The impact of CKD is particularly profound in China, where diabetes has become a predominant factor contributing to the development of end-stage renal disease (5). Recent data from the 2020 Global Burden of Disease Study indicate that China accounted for approximately 132.3 million CKD cases by 2017, representing nearly 20% of the global CKD cases, with diabetes-related CKD contributing to a significant proportion of this figure (6). Given the progressive and often irreversible nature of CKD (7), early identification of at-risk individuals and targeted management strategies are essential to slow disease progression and improve overall patient outcomes.

Among the various metabolic and biochemical markers implicated in CKD pathogenesis, uric acid (UA) and high-density lipoprotein cholesterol (HDL-C) have garnered increasing attention due to their roles in metabolic dysregulation and renal dysfunction (8, 9). Elevated UA levels have been implicated in the development and progression of CKD through multiple mechanisms, including oxidative stress, endothelial dysfunction, systemic inflammation, and renal hemodynamic alterations (10). Specifically, hyperuricemia can induce chronic inflammation by activating the NLRP3 inflammasome and promoting the release of pro-inflammatory cytokines such as IL-1β, which exacerbate renal tubular injury and interstitial fibrosis (11). Additionally, UA contributes to metabolic dysregulation by impairing insulin signaling and promoting insulin resistance (IR), a hallmark of T2DM that further accelerates renal damage through glucotoxicity and lipotoxicity (12). On the other hand, HDL-C, commonly referred to as ‘good cholesterol’, is recognized for its renoprotective properties, which include promoting reverse cholesterol transport, attenuating inflammation, and enhancing endothelial function (13, 14). However, in the context of T2DM, HDL-C functionality may be compromised due to oxidative modifications and glycation, reducing its anti-inflammatory capacity and potentially exacerbating renal injury through lipid accumulation in glomeruli (15). Given the interplay between metabolic abnormalities and renal injury, recent studies have proposed the UA-to-HDL-C ratio (UHR) as a novel composite marker that integrates pro-oxidative and anti-inflammatory pathways, thereby offering a potentially more comprehensive marker of CKD (16, 17). The UHR reflects the balance between UA-driven oxidative stress and inflammation and HDL-C-mediated protection, making it particularly relevant in T2DM, where chronic inflammation and metabolic dysregulation synergistically drive renal pathology (18).

While the individual associations of UA and HDL-C with CKD have been well documented, the clinical relevance of UHR to CKD among diagnosed T2DM patients remains insufficiently explored, particularly in Chinese individuals. Existing research, such as the nationwide cohort study by Liu et al. examining UHR in individuals with abnormal glucose metabolism, has not specifically focused on diagnosed T2DM patients, leaving a critical gap in understanding how UHR relates to CKD in this distinct subgroup (19). Moreover, prior studies have not fully elucidated the specific mechanisms linking UHR to CKD in the context of T2DM-specific pathophysiology, nor have they identified precise UHR thresholds for risk stratification in this population. Given that T2DM is a complex metabolic disorder characterized by chronic low-grade inflammation, IR, and vascular dysfunction (20), these pathophysiological characteristics may amplify the UHR-CKD association by enhancing oxidative stress and inflammatory cascades, potentially involving pathways such as the renin-angiotensin system activation and nuclear factor-κB signaling (21). Thus, a deeper understanding of the UHR-CKD relationship in T2DM patients may uncover novel insights into underlying mechanisms and refine risk prediction models tailored to this population.

To address this knowledge gap, our study utilizes a comprehensive cross-sectional dataset to evaluate the association between UHR and CKD among Chinese individuals with diagnosed T2DM. Specifically, we aim to assess the relationship between UHR and CKD and identify potential UHR cut-off values that could aid in clinical risk stratification. By elucidating this relationship, our findings may help inform future research on personalized intervention strategies for diabetic patients at high risk for CKD progression, ultimately contributing to improved renal outcomes in this vulnerable population.

## 2 Materials and methods

### 2.1 Study subjects

**Figure 1:**
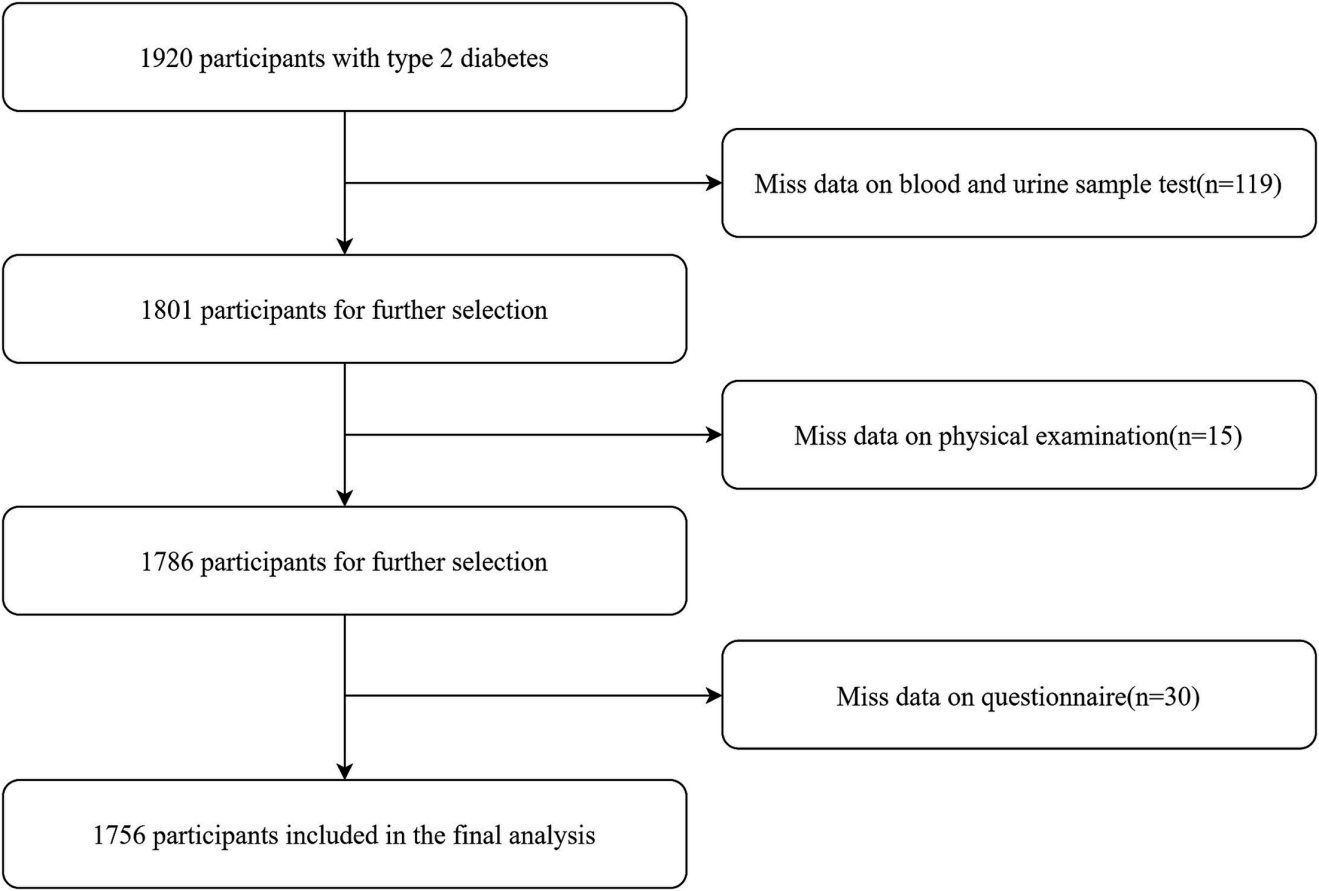
The detailed procedure of participants selection.

The study participants were recruited from an investigation on complications of T2DM, which was conducted from March to November 2018, including local residents with T2DM aged 18 years and above. The selection of participants was conducted through a multi-stage randomized sampling approach. In stage 1, 2 districts and 2 counties were randomly selected from across the province. In stage 2, 4 townships or subdistricts were randomly selected from each of the counties and districts chosen in the first stage. In stage 3, a random sample of 120 individuals per township or subdistrict diagnosed with T2DM was drawn, ensuring a balanced representation across different sexes and age groups. This methodology culminated in the inclusion of 1,920 participants in the study. Additional information concerning the study’s design and methodological details can be found in the published study protocol (22). After applying the exclusion criteria (e.g., lack of questionnaires, physical examinations, blood and urine tests), a total of 164 participants were excluded, resulting in a final sample of 1,756 participants for analysis (Figure 1).

### 2.2 Sample size calculation

The calculation of the sample size was based on the formula: N = deff × μ^2^ × P × (1 − P)/d^2^. In this formula, the parameters were defined as follows: the design effect (deff) was set at 1.2, P represented the prevalence of CKD among Chinese T2DM patients estimated as 0.271 (23), *μ* was set to 1.96, and the relative error (d) was 0.05. Applying these values, the sample size for each stratum was determined to be 365 cases. To account for the stratification of the study population into 4 strata (urban and rural areas, as well as gender) and a non-response rate of 15.0%, the final sample size required for the study was estimated to be 1,718 participants.

### 2.3 Data collection and quality control

In this study, all participants were required to complete an in-person questionnaire survey, undergo physical measurements (e.g., weight, height, blood pressure), and participate in laboratory tests (such as hemoglobin A1c [HbA1c], lipid profile, etc.), renal function tests (urea, creatinine, UA), and the (urine albumin-to-creatinine ratio, among others). All these procedures were carried out by staff from the township health centers, who possessed extensive work experience and had received specialized training for this study project.

Height, weight, and waist circumference were measured using standardized tools: a stadiometer, an electronic scale (HD-390, TANITA, Tokyo, Japan), and a soft retractable measuring tape, respectively. Blood pressure and heart rate were measured using an electronic blood pressure monitor (HBP-1300, OMRON, Kyoto, Japan).

In this survey, fasting blood samples were obtained from all participants following a 10–12 h overnight fast, alongside the collection of first-morning urine specimens. FPG levels were measured by local laboratories at the survey sites that had passed the qualification assessment (glucose oxidase or hexokinase method). The remaining blood and urine specimens were processed at the survey locations, where they were centrifuged, divided into aliquots, and stored following established preservation protocols. These samples were subsequently transported by a logistics service provider appointed by the national project team for further analysis. HbA1c was analyzed via high-performance liquid chromatography (D10, Berkeley, Bio-Rad, USA), lipids (triglycerides [TG], total cholesterol [TC], HDL-C, low-density lipoprotein cholesterol [LDL-C]), UA, blood and urine creatinine levels were determined enzymatically (Cobas C701, Roche, Basel, Switzerland), while urinary albumin was quantified immunoturbidimetrically (Cobas C701, Roche, Basel, Switzerland).

Throughout the entire survey process, strict quality control measures were enforced. Prior to the investigation, all research staff and investigators participated in a comprehensive training session. To ensure adherence to standardized procedures, provincial supervisors randomly re-examined at least 5% of the participants during the investigation phase. Once the survey was completed, all gathered data were uploaded to the national information management platform, where they underwent thorough review and verification.

### 2.4 Outcomes and definitions

The primary outcome of our study was the presence of CKD in T2DM participants, defined by either a reduction in kidney function, as indicated by an estimated glomerular filtration rate (eGFR) of less than 60 mL/min per 1.73 m^2^, or the presence of albuminuria, with a urinary albumin-to-creatinine ratio (UACR) of 30 mg/g or higher (24). The eGFR was computed using the Chronic Kidney Disease Epidemiology Collaboration (CKD-EPI) formula (25). The UHR, expressed as a percentage, was derived by dividing the UA concentration (mg/dL) by the HDL-C level (mg/dL) and then multiplying the result by 100 (26).

Hypertension was identified if participants had systolic blood pressure ≥ 140 mm Hg, diastolic blood pressure ≥ 90 mm Hg, in conjunction with a self-reported history of hypertension diagnosis by healthcare facilities (27). A lipid profile was considered adverse if it met any one of the following thresholds: TC levels at or above 6.22 mmol/L, TG levels at or above 2.26 mmol/L, LDL-C levels at or above 4.14 mmol/L, or HDL-C levels below 1.04 mmol/L (28). Elevated HbA1c and FPG were defined as levels of 7.0% or higher and 7.0 mmol/L or higher, respectively (29).

Educational attainment was segmented into three categories: secondary education or lower, senior high school, and college education or above (29). Age stratification grouped participants into young adults (18–44 years), middle-aged adults (45–59 years), and older adults (60 years and above). Diabetes duration was categorized into two groups: 10 years and below, and over 10 years. Residency was classified as either urban or rural based on the participants’ dwelling locations. Smoking status was determined by current daily or occasional use of cigarettes, while alcohol drinking was noted if subjects had consumed alcohol within the preceding 30 days.

### 2.5 Statistical analysis

Numerical data were expressed in two formats depending on their distribution: means (standard deviations) or medians (IQRs). Categorical data were displayed as frequency (percentage). In the analysis of data exhibiting a normal distribution, group comparisons were conducted using t-tests, whereas the Wilcoxon rank-sum test was applied for data that did not follow a normal distribution. Categorical variables were compared utilizing the chi-square test. Participants were categorized into three groups according to the tertiles of UHR: the first tertile (T1), second tertile (T2) and third tertile (T3). A propensity score matching (PSM) analysis with standardized mean differences (SMD) was conducted to minimize potential confounding and ensure balanced characteristics between the CKD and non-CKD groups. To evaluate the relationship between CKD and influencing factors, multivariable logistic regression models were employed. To evaluate the potential nonlinear association between UHR and CKD, restricted cubic spline (RCS) curves were employed. Three multivariable logistic regression models were constructed: Model 1 was unadjusted analysis model (no covariates included). Model 2 included adjustments for age and sex. Model 3 further adjusted for additional variables such as educational attainment, BMI, unfavorable lipid profile (TC, TG, LDL-C) and glycemic parameters (HbA1c, FPG), hypertension, smoking, alcohol drinking, and diabetes duration. The generalized variance inflation factor (GVIF) for all variables incorporated in the analysis was evaluated to ensure significant multicollinearity was not present in our dataset (all GVIF <2) (Supplementary Table S1). Receiver Operating Characteristic (ROC) curve analysis was performed to evaluate the ability of UHR to distinguish individuals with CKD from those without, as measured by the area under the curve (AUC). The optimal cut-point is determined according to the threshold that maximizes Youden’s Index, which represents the point where sensitivity and specificity are optimally balanced. To assess the incremental predictive value of UHR, net reclassification improvement (NRI) and integrated discrimination improvement (IDI) metrics were employed. Subgroup analyses were conducted to assess the consistency of the association between UHR and CKD across different strata of selected covariates, such as age, sex, BMI, smoking, alcohol drinking status, hypertension, glycemic control and diabetes duration. Additionally, the k-means clustering algorithm was employed to categorize UHR data into distinct clusters during sensitivity analysis (19). The optimal number of clusters was determined using the elbow method, based on the sum of squared errors for varying values of k. The threshold for statistical significance was established at *α* = 0.05. All statistical analyses were executed using R software (version 4.2.1, R Foundation for Statistical Computing, Vienna, Austria) with the packages pROC, MatchIt, tableone, rcssci, rms, PredictABEL, forestplot, metafor, segmented, ggplot2, cluster and nricens.

## 3 Results

### 3.1 Basic characteristics of participants

This cross-sectional study included 1,920 T2DM subjects, with 1,756 participants meeting the inclusion criteria by providing comprehensive demographic and clinical data (Table 1). Among these, 485 individuals (27.62%) were identified with CKD. Of these CKD cases, 70 (14.43%) exhibited a reduced eGFR, 352 (72.58%) had elevated UACR, and 63 (12.99%) presented with both conditions. The study population was stratified based on CKD status, revealing a nearly equal distribution of males (49.89%) and females (50.11%). Participants had an average age of 57.23 years (SD: 10.15 years) and a mean BMI of 24.76 kg/m^2^ (SD: 3.43 kg/m^2^). Comparative analyses demonstrated that individuals with CKD were older, had higher BMI values, elevated UA levels, increased UHR, and more adverse lipid profiles, including higher TC and TG alongside lower HDL-C levels (all *p* < 0.01). Furthermore, glycemic control was significantly poorer in the individuals with CKD, as evidenced by higher HbA1c and FPG levels (all *p* < 0.001). Hypertension prevalence and diabetes duration were also greater among CKD participants (all *p* < 0.001).

**Table 1:** Participants’ basic characteristics by CKD status (*n* = 1,756).

Characteristics	Total (*n* = 1,756)	CKD group (*n* = 485)	Non-CKD group (*n* = 1,271)	*χ*^2^/*t*/*z*	*p*-value
Sex, *n* (%)				0.048^b^	0.827
Female	880 (50.11)	241 (49.69)	639 (50.28)		
Male	876 (49.89)	244 (50.31)	632 (49.72)		
BMI, mean ± SD, kg/m^2^	24.76 ± 3.43	25.43 ± 3.56	24.51 ± 3.35	−5.06^a^	<0.001
Age, mean ± SD, years	57.23 ± 10.15	59.09 ± 10.54	56.52 ± 9.92	−4.77^a^	<0.001
Educational attainment, *n* (%)				8.12^b^	0.017
Secondary education or below	1,541 (87.76)	411 (84.74)	1,130 (88.90)		
Senior high school	171 (9.74)	63 (12.99)	108 (8.50)		
College education or above	44 (2.50)	11 (2.27)	33 (2.60)		
Residence, *n* (%)				1.30^b^	0.255
Urban	881 (50.17)	254 (52.37)	627 (49.33)		
Rural	875 (49.83)	231 (47.63)	644 (50.67)		
Hypertension, *n* (%)	1,099 (62.59)	385 (79.38)	714 (56.18)	80.73^b^	<0.001
TG, median (IQR), mmol/L	1.60 (1.12–2.42)	1.87 (1.30–2.94)	1.51 (1.06–2.26)	47.47^c^	<0.001
TC, mean ± SD, mmol/L	4.65 ± 1.07	4.78 ± 1.29	4.61 ± 0.97	−2.64^a^	0.009
HDL-C, mean ± SD, mmol/L	1.25 ± 0.36	1.18 ± 0.37	1.28 ± 0.35	4.96^a^	<0.001
LDL-C, mean ± SD, mmol/L	2.73 ± 0.90	2.70 ± 1.02	2.75 ± 0.85	0.98^a^	0.325
HbA1c, mean ± SD, %	7.27 ± 1.49	7.61 ± 1.65	7.14 ± 1.40	−5.43^a^	<0.001
FPG, mean ± SD, mmol/L	7.94 ± 2.58	8.54 ± 3.11	7.72 ± 2.31	−5.32^a^	<0.001
UA, mean ± SD, mg/dL	5.63 ± 1.59	6.01 ± 1.87	5.48 ± 1.44	−5.66^a^	<0.001
Diabetes duration (years), *n* (%)				22.88^b^	<0.001
≤10	1,327 (75.57)	328 (67.63)	999 (78.60)		
>10	429 (24.43)	157 (32.37)	272 (21.40)		
UHR	12.92 ± 6.12	14.73 ± 7.22	12.22 ± 5.50	−6.92^a^	<0.001

a Student’s t-test.

b Chi-square test.

c Wilcoxon rank-sum test.

CKD, chronic kidney disease; SD, standard deviation; BMI, body mass index; TC, total cholesterol; IQR, interquartile range; TG, triglycerides; LDL-C, low-density lipoprotein cholesterol; HDL-C, high-density lipoprotein cholesterol; HbA1c, hemoglobin A1c; FPG, fasting plasma glucose; UA, uric acid; UHR, UA to HDL-C ratio.

Table 2 presents the baseline characteristics of participants stratified by UHR tertiles. A comprehensive comparison of demographic, anthropometric, and clinical variables was conducted across these tertiles. Individuals in the upper UHR tertiles were more frequently male and demonstrated elevated BMI, TG levels, and UA concentrations, alongside reduced HDL-C levels (all *p* < 0.05). Additionally, these participants showed a greater prevalence of hypertension, higher rates of urban residence, higher smoking and alcohol drinking rates. Significant disparities were observed among the tertiles in terms of LDL-C levels, diabetes duration, and CKD prevalence (all *p* < 0.05). In contrast, no notable differences were detected in age, educational attainment, TC levels, or the frequency of elevated FPG and HbA1c across the tertiles (all *p* > 0.05).

**Table 2:** Participants’ basic characteristics by UHR tertile (*n* = 1,756).

Variables	Total (*n* = 1,756)	T1 (*n* = 586)	T2 (*n* = 585)	T3 (*n* = 585)	*p*-value
Age (years), Mean ± SD	57.23 ± 10.15	57.82 ± 9.34	57.21 ± 10.18	56.65 ± 10.87	0.144
Sex, *n* (%)					<0.001
Female	880 (50.11)	389 (66.38)	300 (51.28)	191 (32.65)	
Male	876 (49.89)	197 (33.62)	285 (48.72)	394 (67.35)	
Educational attainment, *n* (%)					0.500
Secondary education or lower	1,541 (87.76)	520 (88.74)	517 (88.38)	504 (86.15)	
Senior high school	171 (9.74)	54 (9.22)	51 (8.72)	66 (11.28)	
College education or above	44 (2.51)	12 (2.05)	17 (2.91)	15 (2.56)	
Residence, *n* (%)					0.034
Urban	881 (50.17)	279 (47.61)	283 (48.38)	319 (54.53)	
Rural	875 (49.83)	307 (52.39)	302 (51.62)	266 (45.47)	
BMI, Mean ± SD	24.76 ± 3.43	23.38 ± 3.28	24.94 ± 3.13	25.97 ± 3.38	<0.001
TG, median (IQR), mmol/L	1.60 (1.12,2.42)	1.16 (0.88,1.61)	1.58 (1.20,2.17)	2.38 (1.62,3.75)	<0.001
TC, mean ± SD, mmol/L	4.66 ± 1.07	4.70 ± 0.94	4.68 ± 1.00	4.60 ± 1.25	0.195
LDL-C, mean ± SD, mmol/L	2.73 ± 0.90	2.84 ± 0.87	2.87 ± 0.85	2.48 ± 0.92	<0.001
HDL-C, mean ± SD, mmol/L	1.25 ± 0.36	1.56 ± 0.34	1.24 ± 0.22	0.95 ± 0.21	<0.001
UA (mg/dL)	5.63 ± 1.59	4.33 ± 0.94	5.57 ± 0.98	6.98 ± 1.49	<0.001
Elevated HbA1c, *n* (%)	859 (48.92)	304 (51.88)	279 (47.69)	276 (47.18)	0.211
Elevated FPG, *n* (%)	989 (56.32)	338 (57.68)	327 (55.90)	324 (55.38)	0.708
Hypertension, *n* (%)	1,099 (62.59)	309 (52.73)	361 (61.71)	429 (73.33)	<0.001
Smoking, *n* (%)	436 (24.83)	89 (15.19)	142 (24.27)	205 (35.04)	<0.001
alcohol drinking, *n* (%)	646 (36.79)	199 (33.96)	202 (34.53)	245 (41.88)	0.007
Diabetes duration (years), *n* (%)					<0.001
≤10	1,327 (75.57)	421 (71.85)	462 (78.98)	444 (75.90)	
>10	429 (24.43)	165 (28.15)	123 (21.02)	141 (24.10)	
CKD, *n* (%)	485 (27.62)	131 (22.35)	130 (22.22)	224 (38.29)	<0.001

UHR, UA to HDL-C ratio; T1, first tertile; T2, second tertile; T3, third tertile; BMI, body mass index; TG, triglycerides; TC, total cholesterol; LDL-C, low-density lipoprotein cholesterol; HDL-C, high-density lipoprotein cholesterol; UA, uric acid; HbA1c, hemoglobin A1c; FPG, fasting plasma glucose; CKD, chronic kidney disease.

### 3.2 The relationship between CKD and UHR

To evaluate the relationship between UHR with CKD, multivariable logistic regression models were employed. The odds ratios (ORs) and corresponding 95% confidence intervals (CIs) were estimated across the tertiles of UHR, using the lowest tertile (T1) as the reference category (Table 3). Initially, UHR was analyzed as continuous variable. In the unadjusted model (Model 1), the OR (95%CI) per SD increase in UHR was 1.48 (1.33–1.64). Following the adjustment for age and sex, this OR remained largely unchanged. Subsequent adjustments for additional covariates, including educational attainment, BMI, elevated TC, TG, LDL-C, FPG, HbA1c, hypertension, smoking status, alcohol drinking, and diabetes duration, yielded OR of 1.40 (1.23–1.60). When UHR was analyzed as a categorical variable, the unadjusted analysis demonstrated ORs for CKD of 0.99 (0.75–1.31) for T2 and 2.16 (1.67–2.78) for T3, compared to T1. Following the adjustment for age and sex in Model 2, the ORs exhibited only slight variations. When fully adjusted in Model 3, the ORs for T2 and T3 were 0.93 (0.69–1.25) and 1.82 (1.32–2.50), respectively. All three analytical frameworks demonstrated statistically significant trends (all *p* for trend <0.001).

**Table 3:** Multivariable regression analysis of UHR and its tertiles in relation to CKD (*n* = 1,756).

Characteristics	Model 1		Model 2		Model 3	
	OR (95%CI)	*p*-value	OR (95%CI)	*p*-value	OR (95%CI)	*p*-value
UHR per SD	1.48 (1.33–1.64)	<0.001	1.52 (1.37–1.70)	<0.001	1.40 (1.23–1.60)	<0.001
UHR tertile (range)						
T1 (2.09–9.74)	1.00 (ref)		1.00 (ref)		1.00 (ref)	
T2 (9.75–14.07)	0.99 (0.75–1.31)	0.956	1.01 (0.77–1.34)	0.926	0.93 (0.69–1.25)	0.629
T3 (14.08–55.50)	2.16 (1.67–2.78)	<0.001	2.30 (1.75–3.02)	<0.001	1.82 (1.32–2.50)	<0.001
*p* for trend	1.09 (1.06–1.11)	<0.001	1.09 (1.06–1.12)	<0.001	1.07 (1.04–1.10)	<0.001

UHR, uric acid to high-density lipoprotein cholesterol ratio; CKD, chronic kidney disease; OR, odds ratio; CI, confidence interval; SD, standard deviation; T1, first tertile; T2, second tertile; T3, third tertile; ref, reference.

Model 1: unadjusted analysis (no covariates included); Model 2: adjusted for age, sex.

Model 3: fully adjusted model, incorporating age, sex, education, body mass index, unfavorable lipid profile (total cholesterol, triglyceride, low-density lipoprotein cholesterol) and glycemic parameters (hemoglobin A1c, fasting plasma glucose), hypertension, smoking, alcohol drinking, and diabetes duration.

### 3.3 Linear association between UHR and CKD

**Figure 2:**
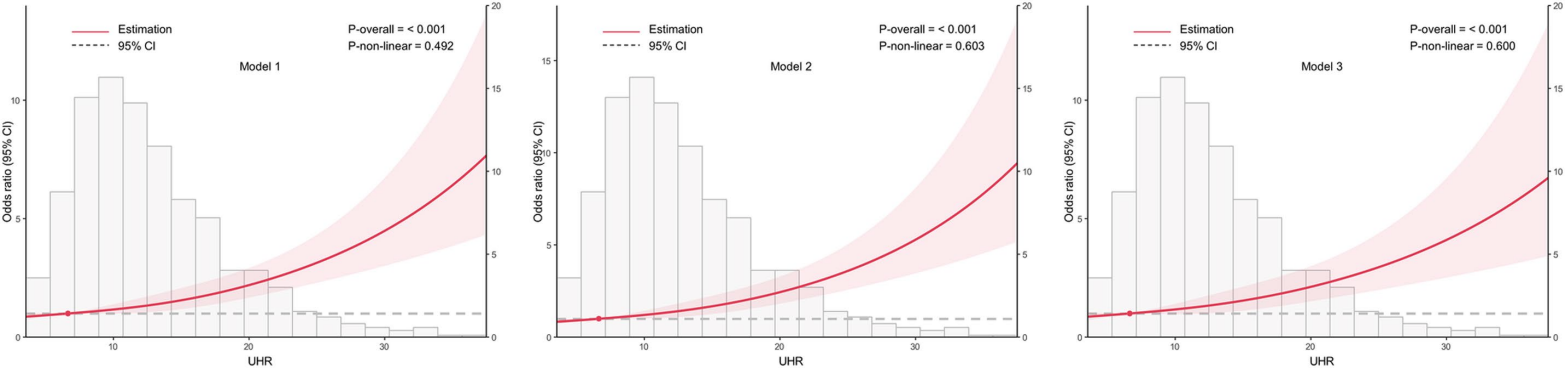
Association between the uric acid to high-density lipoprotein cholesterol ratio (UHR) and chronic kidney disease (CKD) across different models, with 95% confidence intervals (CIs). The figure depicts the relationship between UHR and CKD across three models, considering potential nonlinear associations. Model 1: Unadjusted analysis (no covariates included). Model 2: Adjusted for age and sex, providing a more refined estimate of the association by controlling for these demographic factors. Model 3: Fully adjusted model, incorporating age, sex, education, body mass index, unfavorable lipid profile (total cholesterol, triglyceride, low-density lipoprotein cholesterol) and glycemic parameters (hemoglobin A1c, fasting plasma glucose), hypertension, smoking, alcohol drinking, and diabetes duration. CI, confidence interval; UHR, uric acid to high-density lipoprotein cholesterol ratio.

To examine the dose–response relationship between UHR and CKD, RCS analysis was conducted, as shown in Figure 2. The spline curves were assessed across three separate models: an unadjusted model (Model 1), a partially adjusted model that considered age and sex (Model 2), and a fully adjusted model with additional covariates (Model 3). In all models, a consistent linear dose–response relationship was identified between UHR and CKD (all *p* for nonlinearity >0.05), indicating that increasing UHR levels were associated with a higher likelihood of CKD.

### 3.4 ROC curve assessment

**Figure 3:**
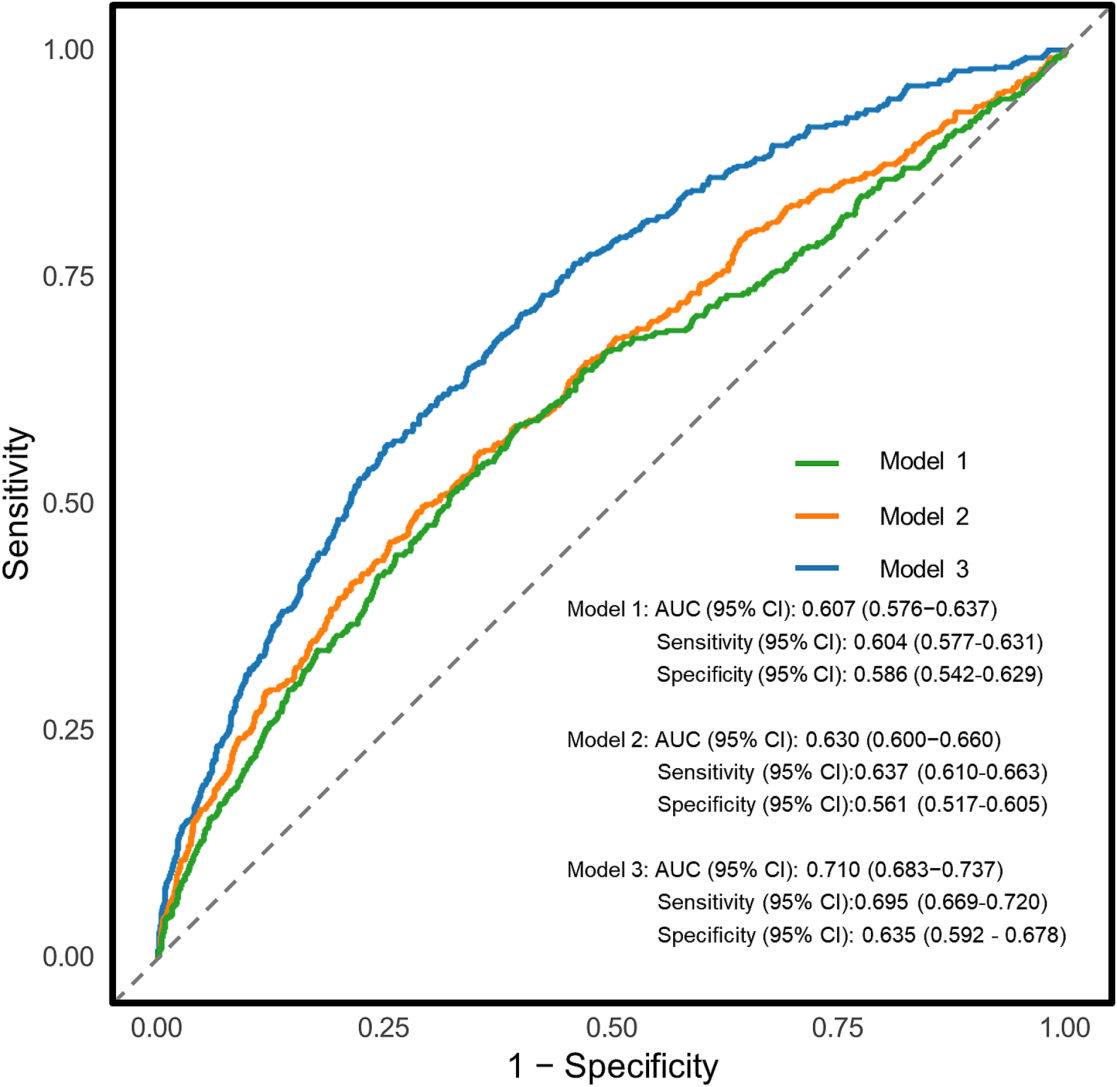
Receiver operating characteristic (ROC) curves for the association between uric acid to high-density lipoprotein cholesterol ratio (UHR) and chronic kidney disease (CKD) in different models. Model 1: Unadjusted analysis (no covariates included); Model 2: Adjusted for age, sex; Model 3: Fully adjusted model, incorporating age, sex, education, body mass index, unfavorable lipid profile (total cholesterol, triglyceride, low-density lipoprotein cholesterol) and glycemic parameters (hemoglobin A1c, fasting plasma glucose), hypertension, smoking, alcohol drinking, and diabetes duration. AUC, area under curve.

**Figure 4:**
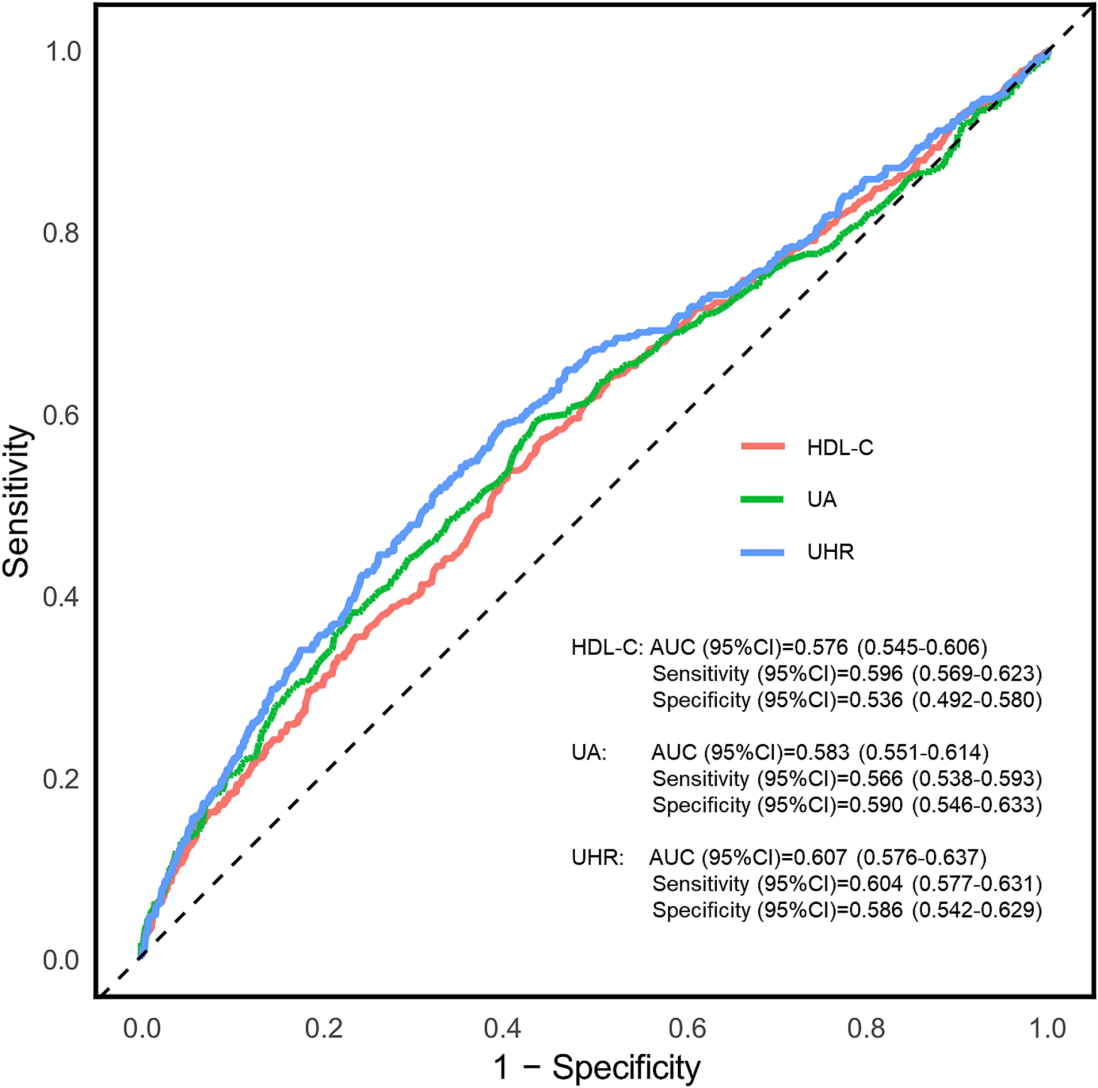
Receiver operating characteristic (ROC) curves for the association between uric acid to high-density lipoprotein cholesterol ratio (UHR), UA and HDL-C with chronic kidney disease (CKD). AUC, area under curve; UA, uric acid; HDL-C, high-density lipoprotein cholesterol; UHR, uric acid to high-density lipoprotein cholesterol ratio.

The diagnostic potential of the UHR for CKD in T2DM patients was assessed through ROC curve analysis (Figure 3). The unadjusted model (Model 1) yielded an AUC (95% CI) of 0.607 (0.576–0.637), demonstrating moderate diagnostic accuracy. The optimal UHR cut-off value was identified as 12.28, with corresponding sensitivity and specificity of 60.4 and 58.6%, respectively, and positive and negative predictive values of 79.3 and 36.1%. After adjusting for age and sex (Model 2), the AUC improved to 0.630 (0.600–0.660). The fully adjusted model (Model 3), incorporating additional covariates such as BMI, education level, unfavorable lipid profiles (TC, TG, LDL-C) and glycemic parameters (FPG, HbA1c), hypertension status, lifestyle factors (smoking, alcohol drinking), and diabetes duration, showed further improvement with an AUC of 0.710 (0.683–0.737), representing moderate diagnostic capability. To assess the incremental predictive value of UHR, we employed NRI and IDI metrics. The results demonstrated that UHR significantly enhanced the predictive performance beyond traditional risk factors (NRI = 20.27%, IDI = 1.56%, both *p* < 0.001). Furthermore, we conducted comparative analyses of UHR against its individual components (UA and HDL-C) using ROC curves (Figure 4). DeLong test revealed that UHR exhibited superior predictive performance compared to either UA or HDL-C alone (both *p* < 0.05), while no significant difference was observed between UA and HDL-C (*p* > 0.05). These findings suggest that the combined UHR metric provides better diagnostic utility than its individual components for identifying CKD in T2DM patients.

### 3.5 Results of subgroup analyses

**Figure 5:**
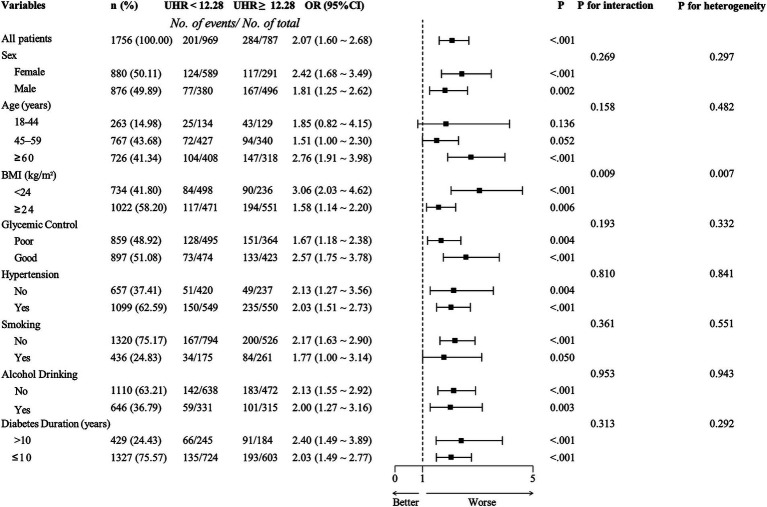
Subgroup analysis of adjusted odds ratios for chronic kidney disease (CKD). Adjusting age, sex, education, body mass index, unfavorable lipid profile (total cholesterol, triglyceride, low-density lipoprotein cholesterol) and glycemic parameters (hemoglobin A1c, fasting plasma glucose), hypertension, smoking, alcohol drinking, and diabetes duration for each subgroup (excluding for its own group). UHR, uric acid to high-density lipoprotein cholesterol ratio; OR, odds ratio; BMI, body mass index.

**Figure 6:**
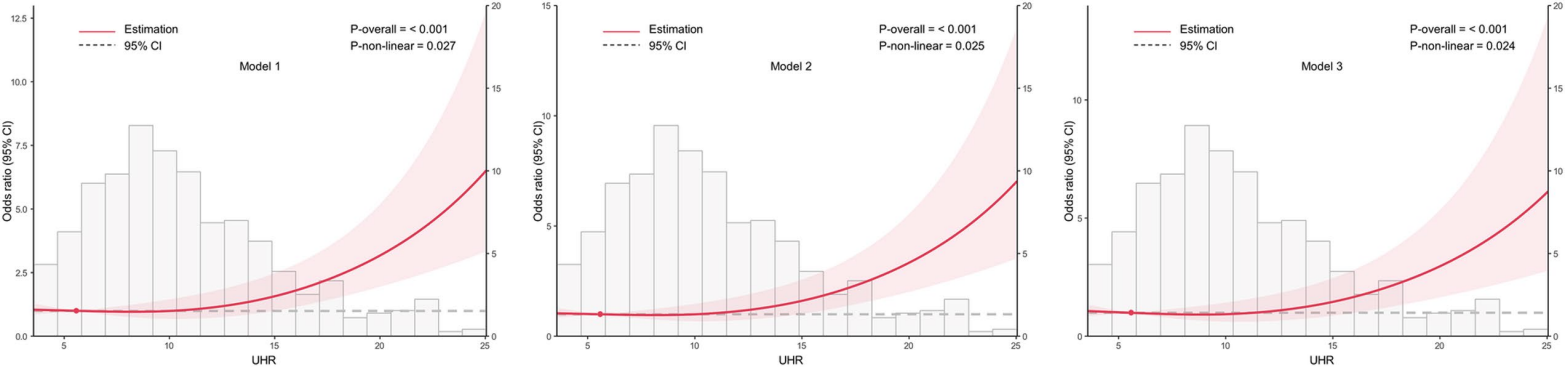
Association between the uric acid to high-density lipoprotein cholesterol ratio (UHR) and chronic kidney disease (CKD) among lower BMI group (BMI < 24 kg/m^2^), with 95% confidence intervals (CIs). The figure depicts the relationship between UHR and CKD across three models, considering potential nonlinear associations. Model 1: Unadjusted analysis (no covariates included). Model 2: Adjusted for age and sex, providing a more refined estimate of the association by controlling for these demographic factors. Model 3: Fully adjusted model, incorporating age, sex, education, unfavorable lipid profile (total cholesterol, triglyceride, low-density lipoprotein cholesterol) and glycemic parameters (hemoglobin A1c, fasting plasma glucose), hypertension, smoking, alcohol drinking, and diabetes duration. CI, confidence interval; UHR, uric acid to high-density lipoprotein cholesterol ratio.

**Figure 7:**
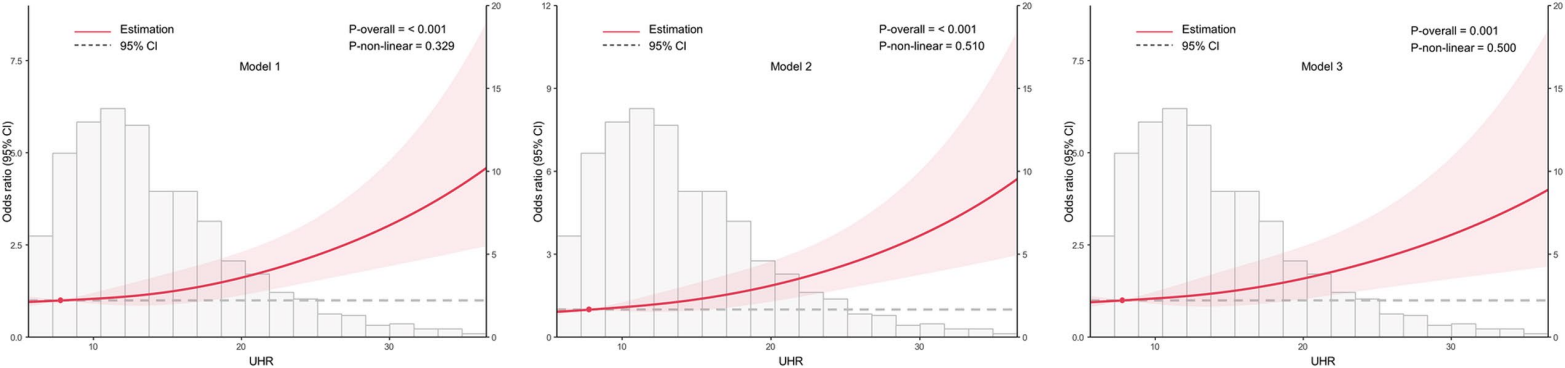
Association between the uric acid to high-density lipoprotein cholesterol ratio (UHR) and chronic kidney disease (CKD) among higher BMI group (BMI ≥ 24 kg/m^2^), with 95% confidence intervals (CIs). The figure depicts the relationship between UHR and CKD across three models, considering potential nonlinear associations. Model 1: Unadjusted analysis (no covariates included). Model 2: Adjusted for age and sex, providing a more refined estimate of the association by controlling for these demographic factors. Model 3: Fully adjusted model, incorporating age, sex, education, unfavorable lipid profile (total cholesterol, triglyceride, low-density lipoprotein cholesterol) and glycemic parameters (hemoglobin A1c, fasting plasma glucose), hypertension, smoking, alcohol drinking, and diabetes duration. CI, confidence interval; UHR, uric acid to high-density lipoprotein cholesterol ratio.

Based on the optimal cut-off value obtained from ROC analysis, the UHR was divided into two groups: <12.28 and ≥12.28. Comprehensive subgroup analyses were performed, encompassing various demographic and clinical factors, including age group (18–44 years, 45–59 years, and ≥60 years), sex (male and female), BMI (<24 kg/m^2^ and ≥24 kg/m^2^), hypertension (yes or no), glycemic control (good or poor), smoking (yes or no), alcohol drinking (yes or no), and diabetes duration (≥10 years or <10 years) (Figure 5). The results indicated that the *p*-values for the association were statistically significant (all *p* < 0.05) in all subgroups except for the 18–44 years age group, the 45–59 years age group, and smoking group, where the associations were not significant (*p* > 0.05). The analysis revealed non-significant interaction and heterogeneity effects across most subgroup comparisons (*p* > 0.05), with the notable exception of BMI, which demonstrated significant interaction (*p* = 0.009) and heterogeneity (*p* = 0.007). These findings indicate that the association between elevated UHR levels and increased CKD risk remained robust and consistent across most demographic and clinical variables examined. Specifically, the OR was higher in individuals with BMI <24 kg/m^2^ compared to those with BMI ≥24 kg/m^2^, indicating a stronger association between UHR and CKD risk in the lower BMI group. To further investigate the association between UHR and CKD, we performed RCS analysis stratified by BMI. The results revealed distinct dose–response patterns based on BMI categories. In participants with lower BMI (<24 kg/m^2^), a nonlinear relationship was consistently observed between UHR and CKD across all models, including crude, partially adjusted, and fully adjusted models (all *p* for nonlinearity <0.05) (Figure 6). Conversely, among participants with higher BMI (≥24 kg/m^2^), a linear association was consistently demonstrated between UHR and CKD in all models (all p for nonlinearity >0.05) (Figure 7). These findings suggest that BMI significantly modifies the nature of the UHR-CKD relationship.

### 3.6 Sensitivity analyses

To enhance the reliability and validity of our research findings, we conducted a series of comprehensive sensitivity analyses. First, we employed PSM to mitigate potential confounding factors and strengthen the robustness of our results. As shown in Supplementary Table S2, the distribution of covariates between the CKD and non-CKD groups was significantly improved after PSM, with all SMDs reduced to below 0.1, indicating a well-balanced matched population. This confirmed the effectiveness of PSM in creating comparable study groups. Subsequently, we performed multivariable logistic regression analyses on the matched dataset to evaluate the association between UHR (both as a continuous variable and in tertiles) and CKD risk (Supplementary Table S3). These analyses were conducted across three models: crude, partially adjusted, and fully adjusted. The results from the matched cohort analysis were consistent with our initial findings prior to PSM, further reinforcing the stability and reliability of our primary conclusions. Additionally, we utilized the k-means clustering algorithm to categorize UHR data into distinct clusters. Supplementary Figure S1 illustrates a clear elbow point was at k = 4, indicating that dividing UHR levels into four clusters provided an optimal balance between accuracy and computational efficiency. Specifically: cluster 1 (UHR range: 2.09–10.34), cluster 2 (UHR range: 10.34–16.28), cluster 3 (UHR range: 16.30–25.68) and cluster 4 (UHR range: 25.82–55.50). We then applied multivariate logistic regression models to analyze the relationship between UHR clusters and CKD (Supplementary Table S4). In the fully adjusted model 3, the odds ratios (ORs) for cluster 2, cluster 3, and cluster 4 were 1.17 (95% CI: 0.89–1.55), 1.93 (95% CI: 1.37–2.73), and 3.35 (95% CI: 1.84–6.11), respectively. All three clusters demonstrated statistically significant trends (all p for trend <0.001), consistent with the relationship observed between UHR tertiles and CKD. Finally, given the nonlinear relationship between UHR and CKD in the lower BMI group (BMI < 24 kg/m^2^) as indicated by RCS analysis, we conducted a piecewise logistic regression model to further explore this relationship. As shown in Supplementary Table S4, the piecewise model provided a significantly better fit than the standard logistic regression model (*p* = 0.012). The analysis revealed a threshold effect at a UHR of 5.85. Below this threshold, higher UHR was associated with a reduced risk of CKD, whereas above this threshold, higher UHR was associated with an increased risk of CKD.

## 4 Discussion

This study identified a significant positive correlation between the UHR and CKD in a Chinese population aged 18 years and older with T2DM. Our analysis revealed that individuals with higher UHR levels exhibited a greater likelihood of CKD, with this relationship persisting even after adjusting for multiple confounders. Notably, a linear dose–response relationship was observed, suggesting that increasing UHR levels consistently correlated with an elevated CKD risk. Furthermore, subgroup analyses demonstrated that this association remained robust across most demographic and clinical variables, except for the 18–44 and 45–59 age groups and smokers. Importantly, BMI was identified as a significant effect modifier, with a nonlinear relationship observed in individuals with lower BMI, whereas a linear association persisted among those with higher BMI. ROC curve analysis further established the diagnostic potential of UHR, with an optimal cut-off value of 12.28 providing moderate predictive accuracy. Sensitivity analyses, including PSM and clustering approaches, reinforced the stability and reliability of our primary findings. These results support the clinical relevance of UHR as a potential biomarker for CKD risk assessment in T2DM patients.

The positive linear association between the UHR and CKD in T2DM populations may be driven by a combination of metabolic dysregulation, chronic inflammation, and vascular dysfunction. Given that UHR reflects both elevated UA and reduced HDL-C, it serves as a composite marker of metabolic stress and systemic inflammation (30), both of which are key contributors to CKD progression in diabetic patients (31). Uric acid-induced renal injury is particularly relevant in T2DM due to the already compromised renal function in these patients (32). Elevated UA contributes to glomerular hypertension, oxidative stress, and endothelial dysfunction, all of which accelerate renal injury (33, 34). In diabetic kidneys, UA further promotes NLRP3 inflammasome activation, leading to increased secretion of pro-inflammatory cytokines such as IL-1β and TNF-α, which drive tubulointerstitial fibrosis (35–37). Additionally, uric acid impairs mitochondrial function in renal tubular cells, exacerbating diabetic kidney disease through epithelial-to-mesenchymal transition (EMT) and fibrosis (38, 39). Recent research has further elucidated that UA activates the xanthine oxidase pathway, generating reactive oxygen species (ROS) that exacerbate podocyte injury and glomerulosclerosis in T2DM models (40). Beyond these established pathways, UHR may influence CKD development through additional mechanisms, such as the upregulation of toll-like receptor 4 signaling, which amplifies inflammatory responses in renal tissues, or by promoting microvascular damage via the inhibition of nitric oxide bioavailability (41). Compared to other inflammatory markers like C-reactive protein or interleukin-6, UHR offers a unique advantage by integrating both pro-oxidative (UA) and protective (HDL-C) components, providing a more holistic reflection of metabolic-inflammatory imbalance in T2DM-related CKD.

Low HDL-C levels further increase CKD risk in T2DM patients by impairing its normal anti-inflammatory, antioxidant, and endothelial-protective functions (42–44). HDL plays a crucial role in reverse cholesterol transport, preventing lipid accumulation in renal tissues (45, 46). However, in diabetic patients, HDL dysfunction is common, leading to increased oxidative stress, vascular injury, and chronic low-grade inflammation—all of which contribute to CKD progression (47, 48).

IR and metabolic dysfunction may further mediate the link between UHR and CKD in T2DM. Both elevated UA and low HDL-C are strongly associated with IR, which in turn contributes to glomerular hyperfiltration, podocyte injury, and lipid accumulation in renal tubules, leading to progressive nephron loss (49, 50). Additionally, IR activates inflammatory pathways and the renin-angiotensin-aldosterone system, worsening hypertension and accelerating diabetic kidney disease (51, 52). Finally, systemic vascular dysfunction plays a central role in CKD development among T2DM patients (53). Given these multiple mechanisms, UHR may serve as a valuable tool for early CKD risk stratification in diabetic patients, potentially guiding clinical decisions such as intensified monitoring or targeted interventions to mitigate renal decline. For instance, patients with elevated UHR could benefit from early uric acid-lowering therapies (e.g., allopurinol) or HDL-C enhancing strategies (e.g., niacin or lifestyle modifications), though clinical trials are needed to validate these approaches.

The finding that lower BMI strengthens the association between UHR and CKD suggests that BMI plays a critical role in modulating the underlying pathophysiology linking UHR to renal dysfunction (54). In individuals with lower BMI, the UHR-CKD relationship may be more pronounced due to reduced visceral fat buffering of UA, leading to higher circulating UA levels and impaired renal excretion (55). This could reflect a state of unopposed UA accumulation, where the absence of visceral fat allows UA-driven oxidative stress and inflammation to exert a more direct impact on renal tissue (56). Alternatively, lower BMI might indicate sarcopenia or malnutrition in T2DM patients, which could exacerbate oxidative stress and inflammation, further amplifying UHR’s detrimental effects on kidney function (57). Our study’s RCS analysis provides further insight into this modulation, revealing a nonlinear UHR-CKD relationship in the lower BMI group, with a threshold effect at UHR 5.85. Below this threshold, lower UHR levels may reflect insufficient UA-driven metabolic stress to trigger CKD, possibly due to preserved renal clearance or reduced inflammatory burden. However, above this threshold, the sharp increase in CKD risk likely stems from unopposed UA accumulation, which activates pathways such as the NLRP3 inflammasome and xanthine oxidase, driving reactive oxygen species production and tubulointerstitial damage (58). This nonlinearity suggests a tipping point where UA’s pro-oxidative effects overwhelm HDL-C’s protective capacity in leaner T2DM patients. In contrast, the higher BMI group exhibited a linear UHR-CKD relationship, likely reflecting a chronic, obesity-driven inflammatory milieu. In these individuals, visceral fat amplifies IR and lipid dysregulation, synergizing with UHR to progressively worsen CKD without a distinct threshold (59). Additionally, adipocyte-derived adipokines may enhance endothelial dysfunction and glomerular hypertension, sustaining a steady UHR-CKD link (60). For leaner individuals, sarcopenia could further exacerbate the UHR-CKD relationship by reducing muscle mass-dependent UA metabolism, while in obese patients, the dominant role of visceral fat and systemic inflammation may overshadow UHR’s independent effects. These BMI-specific mechanisms highlight the need for tailored interventions. Lean patients may benefit from early uric acid-lowering therapies below critical thresholds to prevent the tipping point where UA-driven damage becomes irreversible. In contrast, obese patients might require combined anti-inflammatory and lipid-modulating strategies to disrupt the linear progression of CKD.

The lack of significant associations in younger patients and smokers is another notable finding that requires further investigation. Age-related metabolic variations and smoking-related mechanisms may influence the ability of UHR to distinguish CKD status in these subgroups. In younger individuals, renal function may be less affected by chronic inflammatory and metabolic stressors that elevate uric acid and impair HDL-C metabolism, resulting in weaker associations with CKD (61). Younger individuals typically have better renal reserve and fewer cumulative metabolic insults, which may mask the impact of UHR on kidney function. As individuals age, these metabolic disturbances, including oxidative stress, chronic inflammation, and IR, become more pronounced, thereby strengthening the link between UHR and kidney damage (62). This suggests that UHR may be a more reliable biomarker for CKD risk in older populations, where metabolic dysregulation and renal vulnerability are more evident. Regarding smoking, while it is a well-known risk factor for CKD, its interaction with UHR may be more complex. Smoking induces systemic inflammation, oxidative stress, and endothelial dysfunction, all of which contribute to kidney injury (63). However, the effect of smoking on UHR and CKD risk may vary depending on factors such as smoking duration, intensity, and the presence of comorbidities like hypertension or diabetes. For instance, smoking may alter uric acid metabolism and HDL-C functionality in ways that are not fully captured by UHR alone. Smokers often exhibit higher levels of oxidative stress and inflammation, which could potentially overwhelm the protective effects of HDL-C, making UHR less predictive of CKD in this subgroup (64). These findings have important implications for clinical practice. In younger individuals, UHR may not be as effective in predicting CKD risk due to their relatively preserved renal function and lower burden of metabolic disturbances. Clinicians should consider alternative biomarkers or risk stratification tools for younger patients, particularly those without significant metabolic comorbidities. For smokers, the complex interplay between smoking-related mechanisms and UHR suggests that UHR alone may not be sufficient to assess CKD risk in this population. Instead, a more comprehensive evaluation that includes smoking history, intensity, and duration, along with other metabolic and inflammatory markers, may be necessary to accurately predict CKD risk in smokers.

Our findings align with and extend the existing literature on UHR and CKD. A key comparison can be made with Liu et al., a nationwide cohort study using CHARLS data, which found a positive correlation between UHR and CKD risk in 3,902 Chinese adults with abnormal glucose metabolism (19). Both studies confirm UHR’s association with CKD, but from different perspectives: Liu et al. employed a longitudinal design tracking UHR changes over time (2011–2015), reporting ORs for CKD incidence increasing from 1.08 to 2.13 across UHR classes, while our cross-sectional study provides a snapshot of UHR’s association with prevalent CKD (ORs significant across tertiles). Our study found that per SD increase in UHR was associated with a 40% higher odds of CKD (OR = 1.40, 95% CI: 1.23–1.60) after adjusting for potential covariates, while Liu et al.’s study indicated that each 1-SD rise in cumulative UHR levels, the likelihood of developing CKD increased by 32%. Other similarities include the linear UHR-CKD relationship and robustness across sensitivity analyses. However, differences arise in study design, population focus and outcomes. Liu et al.’s study was longitudinal, could establish temporal relationships and assess causality, while our cross-sectional analysis provides a comprehensive snapshot of UHR-CKD associations, allowing for detailed subgroup analysis and potential effect modifiers. Together, these studies complement each other by providing both immediate associations and insights into the progression of CKD over time, highlighting the need for further prospective studies to validate UHR as a predictive biomarker for CKD in high-risk populations. Other studies, such as Wang et al. on contrast-induced acute kidney injury (65) and Cheng et al. on a health check-up population (17), also support UHR’s renal risk association, though they differ in context (acute kidney injury) and population (older population in the united states), highlighting UHR’s broad applicability but T2DM-specific gaps our study addresses.

The strengths of this study include the following aspects. First, to our knowledge, it is the first to investigate the relationship between UHR and CKD in a well-defined population of Chinese patients with T2DM, offering important insights into this association within a high-risk population. Second, we utilized standardized data collection protocols, ensuring the reliability of our findings.

However, a more critical evaluation of limitations reveals areas for caution. Firstly, the study sample was restricted to individuals with T2DM from Eastern China, potentially limiting generalizability to other regions or ethnicities with differing dietary, genetic, or lifestyle factors. Secondly, despite comprehensive adjustments, residual confounding from unmeasured variables (e.g., genetic predisposition, dietary purine intake) cannot be excluded. Thirdly, the absence of medication data, such as diuretics or urate-lowering agents, is a significant limitation, as these could directly influence UA levels and thus UHR, potentially overestimating its association with CKD. Lastly, the cross-sectional design precludes causality inference, as it captures only a single time point, potentially missing dynamic UHR changes or reverse causation (e.g., CKD altering UHR). This design limitation may underestimate the true strength of the UHR-CKD relationship, particularly if longitudinal trends are more predictive. Potential confounders, such as socioeconomic status or physical activity, could bias the results. The direction of this bias, either toward or away from the null, depends on their distribution.

## 5 Conclusion

This study underscores the association between UHR, a marker of systemic inflammation, and CKD prevalence in individuals with T2DM, revealing a clear dose–response relationship. These findings position UHR as a potentially valuable parameter for CKD risk assessment in diabetic patients, with important implications for clinical practice. Specifically, UHR could be incorporated into routine metabolic panels for T2DM patients, with the identified cut-off of 12.28 serving as a trigger for enhanced renal monitoring (e.g., eGFR, albuminuria) or early interventions such as uric acid reduction or HDL-C optimization. Building on Liu et al.’s study, this research further elucidates UHR’s role in CKD pathogenesis, emphasizing its potential as both a biomarker and therapeutic target. Future studies should prioritize randomized controlled trials to explore interventions targeting UHR-related pathways, such as anti-inflammatory or antioxidant therapies, to evaluate their efficacy in preventing CKD progression in T2DM. Additionally, integrating UHR into risk stratification models could enable more personalized treatment strategies, tailoring interventions to individual metabolic and inflammatory profiles.

## Data Availability

The raw data supporting the conclusions of this article will be made available by the authors without undue reservation.

## References

[1] Kovesdy CP Isaman D Petruski-Ivleva N Fried L Blankenburg M Gay A. Chronic kidney disease progression among patients with type 2 diabetes identified in us administrative claims: a population cohort study. Clin Kidney J. (2021) 14:1657–64. doi: 10.1093/ckj/sfaa200, PMID: 34084461 PMC8162850

[2] Hoogeveen EK. The epidemiology of diabetic kidney disease. Kidney Dial. (2022) 2:433–42. doi: 10.3390/kidneydial2030038

[3] Kovesdy CP. Epidemiology of chronic kidney disease: an update 2022. Kidney Int Suppl (2011). (2022) 12:7–11. doi: 10.1016/j.kisu.2021.11.003 35529086 PMC9073222

[4] Jager KJ Kovesdy C Langham R Rosenberg M Jha V Zoccali C. A single number for advocacy and communication-worldwide more than 850 million individuals have kidney diseases. Nephrol Dial Transplant. (2019) 34:1803–5. doi: 10.1093/ndt/gfz174, PMID: 31566230

[5] Pan X Lin X Huang X Xu J Ye L Zhang T. The burden of diabetes-related chronic kidney disease in China from 1990 to 2019. Front Endocrinol (Lausanne). (2022) 13:892860. doi: 10.3389/fendo.2022.892860, PMID: 35784566 PMC9240757

[6] GBD Chronic Kidney Disease Collaboration. Global, regional, and national burden of chronic kidney disease, 1990-2017: a systematic analysis for the global burden of disease study 2017. Lancet. (2020) 395:709–33. doi: 10.1016/S0140-6736(20)30045-3, PMID: 32061315 PMC7049905

[7] Wang K Liu Q Tang M Qi G Qiu C Huang Y. Chronic kidney disease-induced muscle atrophy: molecular mechanisms and promising therapies. Biochem Pharmacol. (2023) 208:115407. doi: 10.1016/j.bcp.2022.115407, PMID: 36596414

[8] Xu L Li D Song Z Liu J Zhou Y Yang J. The association between monocyte to high-density lipoprotein cholesterol ratio and chronic kidney disease in a Chinese adult population: a cross-sectional study. Ren Fail. (2024) 46:2331614. doi: 10.1080/0886022X.2024.2331614, PMID: 38522954 PMC10962299

[9] Ghang B Park J Lee JS Lim JS Kim H Liew D. Post-hoc analysis of the cares trial suggests delayed progression of chronic kidney disease in patients with gout during urate-lowering therapy. Kidney Int. (2024) 107:521–9. doi: 10.1016/j.kint.2024.10.022, PMID: 39551133

[10] Du L Zong Y Li H Wang Q Xie L Yang B. Hyperuricemia and its related diseases: mechanisms and advances in therapy. Signal Transduct Target Ther. (2024) 9:212. doi: 10.1038/s41392-024-01916-y, PMID: 39191722 PMC11350024

[11] Xiong W Meng XF Zhang C. Nlrp3 inflammasome in metabolic-associated kidney diseases: an update. Front Immunol. (2021) 12:714340. doi: 10.3389/fimmu.2021.714340, PMID: 34305953 PMC8297462

[12] Giri B Dey S Das T Sarkar M Banerjee J Dash SK. Chronic hyperglycemia mediated physiological alteration and metabolic distortion leads to organ dysfunction, infection, cancer progression and other pathophysiological consequences: an update on glucose toxicity. Biomed Pharmacother. (2018) 107:306–28. doi: 10.1016/j.biopha.2018.07.157, PMID: 30098549

[13] Kon V Yang HC Smith LE Vickers KC Linton MF. High-density lipoproteins in kidney disease. Int J Mol Sci. (2021) 22:22. doi: 10.3390/ijms22158201, PMID: 34360965 PMC8348850

[14] von Eckardstein A Nordestgaard BG Remaley AT Catapano AL. High-density lipoprotein revisited: biological functions and clinical relevance. Eur Heart J. (2023) 44:1394–407. doi: 10.1093/eurheartj/ehac605, PMID: 36337032 PMC10119031

[15] Femlak M Gluba-Brzozka A Cialkowska-Rysz A Rysz J. The role and function of HDL in patients with diabetes mellitus and the related cardiovascular risk. Lipids Health Dis. (2017) 16:207. doi: 10.1186/s12944-017-0594-3, PMID: 29084567 PMC5663054

[16] Kolahi AR Mansoori A Sahranavard T Miri MS Feizi S Esmaily H. Serum uric acid to high-density lipoprotein ratio as a novel indicator of inflammation is correlated with the presence and severity of metabolic syndrome: a large-scale study. Endocrinol Diabetes Metab. (2023) 6:e446. doi: 10.1002/edm2.446, PMID: 37605374 PMC10638626

[17] Cheng Y Zhang H Zheng H Yin H Wang Y Wang H. Association between serum uric acid/HDL-cholesterol ratio and chronic kidney disease: a cross-sectional study based on a health check-up population. BMJ Open. (2022) 12:e66243. doi: 10.1136/bmjopen-2022-066243, PMID: 36581406 PMC9806076

[18] Tsalamandris S Antonopoulos AS Oikonomou E Papamikroulis GA Vogiatzi G Papaioannou S. The role of inflammation in diabetes: current concepts and future perspectives. Eur Cardiol. (2019) 14:50–9. doi: 10.15420/ecr.2018.33.1, PMID: 31131037 PMC6523054

[19] Liu Q Zheng D Shen X Jin J He Q. Association between uric acid to high-density lipoprotein cholesterol ratio and chronic kidney disease among Chinese middle-aged and older adults with abnormal glucose metabolism: a nationwide cohort study. Int Urol Nephrol. (2024) 57:1297–309. doi: 10.1007/s11255-024-04308-x, PMID: 39623196

[20] Galicia-Garcia U Benito-Vicente A Jebari S Larrea-Sebal A Siddiqi H Uribe KB. Pathophysiology of type 2 diabetes mellitus. Int J Mol Sci. (2020) 21:6275. doi: 10.3390/ijms21176275, PMID: 32872570 PMC7503727

[21] Wang N Zhang C. Oxidative stress: a culprit in the progression of diabetic kidney disease. Antioxidants (Basel). (2024) 13:13. doi: 10.3390/antiox13040455, PMID: 38671903 PMC11047699

[22] Hou XH Wang LM Chen SY Liang YB Zhang M Huang ZJ. Data resource profile: a protocol of China national diabetic chronic complications study. Biomed Environ Sci. (2022) 35:633–40. doi: 10.3967/bes2022.078, PMID: 35945178

[23] Guo K Zhang L Zhao F Lu J Pan P Yu H. Prevalence of chronic kidney disease and associated factors in chinese individuals with type 2 diabetes: cross-sectional study. J Diabetes Complicat. (2016) 30:803–10. doi: 10.1016/j.jdiacomp.2016.03.020, PMID: 27068269

[24] Wang L Xu X Zhang M Hu C Zhang X Li C. Prevalence of chronic kidney disease in China: results from the sixth China chronic disease and risk factor surveillance. JAMA Intern Med. (2023) 183:298–310. doi: 10.1001/jamainternmed.2022.6817, PMID: 36804760 PMC9941971

[25] Giavarina D Husain-Syed F Ronco C. Clinical implications of the new equation to estimate glomerular filtration rate. Nephron Clin Pract. (2021) 145:508–12. doi: 10.1159/000516638, PMID: 34120119

[26] Zhou X Xu J. Association between serum uric acid-to-high-density lipoprotein cholesterol ratio and insulin resistance in an American population: a population-based analysis. J Diabetes Investig. (2024) 15:762–71. doi: 10.1111/jdi.14170, PMID: 38407574 PMC11143423

[27] Wang JG. Chinese hypertension guidelines. Pulse (Basel). (2015) 3:14–20. doi: 10.1159/000382025, PMID: 26587453 PMC4646151

[28] Wang X Qiu M Cheng Z Ji X Chen J Zhu H. Efficacy and safety of ongericimab in Chinese patients with primary hypercholesterolemia and mixed dyslipidemia. J Am Heart Assoc. (2024) 13:e33669. doi: 10.1161/JAHA.123.033669, PMID: 38818934 PMC11255649

[29] Chen XY Fang L Zhang J Zhong JM Lin JJ Lu F. The association of body mass index and its interaction with family history of dyslipidemia towards dyslipidemia in patients with type 2 diabetes: a cross-sectional study in Zhejiang province, China. Front Public Health. (2023) 11:1188212. doi: 10.3389/fpubh.2023.1188212, PMID: 37255759 PMC10225544

[30] Liu M Cao B Luo Q Song Y Liu K Wu D. Association between serum uric acid-to-high-density lipoprotein cholesterol ratio and metabolic dysfunction-associated steatotic liver disease among Chinese children with obesity. Front Endocrinol (Lausanne). (2024) 15:1474384. doi: 10.3389/fendo.2024.1474384, PMID: 39845880 PMC11750666

[31] Charlton A Garzarella J Jandeleit-Dahm K Jha JC. Oxidative stress and inflammation in renal and cardiovascular complications of diabetes. Biology (Basel). (2020) 10:10. doi: 10.3390/biology10010018, PMID: 33396868 PMC7830433

[32] Srivastava A Kaze AD McMullan CJ Isakova T Waikar SS. Uric acid and the risks of kidney failure and death in individuals with CKD. Am J Kidney Dis. (2018) 71:362–70. doi: 10.1053/j.ajkd.2017.08.017, PMID: 29132945 PMC5828916

[33] Park JH Jo YI Lee JH. Renal effects of uric acid: hyperuricemia and hypouricemia. Korean J Intern Med. (2020) 35:1291–304. doi: 10.3904/kjim.2020.410, PMID: 32872730 PMC7652664

[34] Amiya E. Link between hyperuricemia, renal dysfunction, and hypertension. J Clin Hypertens (Greenwich). (2021) 23:2078–9. doi: 10.1111/jch.14389, PMID: 34806304 PMC8696238

[35] Wan J Liu D Pan S Zhou S Liu Z. Nlrp3-mediated pyroptosis in diabetic nephropathy. Front Pharmacol. (2022) 13:998574. doi: 10.3389/fphar.2022.998574, PMID: 36304156 PMC9593054

[36] Han Y Xu X Tang C Gao P Chen X Xiong X. Reactive oxygen species promote tubular injury in diabetic nephropathy: the role of the mitochondrial ros-txnip-nlrp3 biological axis. Redox Biol. (2018) 16:32–46. doi: 10.1016/j.redox.2018.02.013, PMID: 29475133 PMC5842313

[37] Jin J Zhang M. Exploring the role of nlrp3 inflammasome in diabetic nephropathy and the advancements in herbal therapeutics. Front Endocrinol (Lausanne). (2024) 15:1397301. doi: 10.3389/fendo.2024.1397301, PMID: 39104818 PMC11299242

[38] Wang Y Jin M Cheng CK Li Q. Tubular injury in diabetic kidney disease: molecular mechanisms and potential therapeutic perspectives. Front Endocrinol (Lausanne). (2023) 14:1238927. doi: 10.3389/fendo.2023.1238927, PMID: 37600689 PMC10433744

[39] Qi R Yang C. Renal tubular epithelial cells: the neglected mediator of tubulointerstitial fibrosis after injury. Cell Death Dis. (2018) 9:1126. doi: 10.1038/s41419-018-1157-x, PMID: 30425237 PMC6233178

[40] Korsmo HW Ekperikpe US Daehn IS. Emerging roles of xanthine oxidoreductase in chronic kidney disease. Antioxidants (Basel). (2024) 13:13. doi: 10.3390/antiox13060712, PMID: 38929151 PMC11200862

[41] Liu M Zen K. Toll-like receptors regulate the development and progression of renal diseases. Kidney Dis (Basel). (2021) 7:14–23. doi: 10.1159/000511947, PMID: 33614730 PMC7879300

[42] Xu Z Yang S Cui L. Understanding the heterogeneity and dysfunction of hdl in chronic kidney disease: insights from recent reviews. BMC Nephrol. (2024) 25:400. doi: 10.1186/s12882-024-03808-3, PMID: 39511510 PMC11542271

[43] Bonilha I Zimetti F Zanotti I Papotti B Sposito AC. Dysfunctional high-density lipoproteins in type 2 diabetes mellitus: molecular mechanisms and therapeutic implications. J Clin Med. (2021) 10:10. doi: 10.3390/jcm10112233, PMID: 34063950 PMC8196572

[44] Ebtehaj S Gruppen EG Parvizi M Tietge U Dullaart R. The anti-inflammatory function of HDL is impaired in type 2 diabetes: role of hyperglycemia, paraoxonase-1 and low grade inflammation. Cardiovasc Diabetol. (2017) 16:132. doi: 10.1186/s12933-017-0613-8, PMID: 29025405 PMC5639738

[45] Rysz J Gluba-Brzozka A Rysz-Gorzynska M Franczyk B. The role and function of hdl in patients with chronic kidney disease and the risk of cardiovascular disease. Int J Mol Sci. (2020) 21:21. doi: 10.3390/ijms21020601, PMID: 31963445 PMC7014265

[46] Duan Y Gong K Xu S Zhang F Meng X Han J. Regulation of cholesterol homeostasis in health and diseases: from mechanisms to targeted therapeutics. Signal Transduct Target Ther. (2022) 7:265. doi: 10.1038/s41392-022-01125-5, PMID: 35918332 PMC9344793

[47] Baaten C Vondenhoff S Noels H. Endothelial cell dysfunction and increased cardiovascular risk in patients with chronic kidney disease. Circ Res. (2023) 132:970–92. doi: 10.1161/CIRCRESAHA.123.321752, PMID: 37053275 PMC10097498

[48] Pavanello C Ossoli A. HDL and chronic kidney disease. Atheroscler Plus. (2023) 52:9–17. doi: 10.1016/j.athplu.2023.04.001, PMID: 37193017 PMC10182177

[49] Liu R Li Z Zhang Y Du M Wang X Zhang S. Association of serum uric acid with indices of insulin resistance: proposal of a new model with reference to gender differences. Diabetes Metab Syndr Obes. (2024) 17:3783–93. doi: 10.2147/DMSO.S481233, PMID: 39430137 PMC11491090

[50] Tonneijck L Muskiet MH Smits MM van Bommel EJ Heerspink HJ van Raalte DH. Glomerular hyperfiltration in diabetes: mechanisms, clinical significance, and treatment. J Am Soc Nephrol. (2017) 28:1023–39. doi: 10.1681/ASN.2016060666, PMID: 28143897 PMC5373460

[51] Amorim RG Guedes G Vasconcelos S Santos J. Kidney disease in diabetes mellitus: cross-linking between hyperglycemia, redox imbalance and inflammation. Arq Bras Cardiol. (2019) 112:577–87. doi: 10.5935/abc.20190077, PMID: 31188964 PMC6555585

[52] Zhang Z Zhao L Zhou X Meng X Zhou X. Role of inflammation, immunity, and oxidative stress in hypertension: new insights and potential therapeutic targets. Front Immunol. (2022) 13:1098725. doi: 10.3389/fimmu.2022.1098725, PMID: 36703963 PMC9871625

[53] Hesp AC Smits MM van Bommel EJM Muskiet MHA Tonneijck L Nieuwdorp M. Kidney hemodynamic profile and systemic vascular function in adults with type 2 diabetes: analysis of three clinical trials. J Diabetes Complicat. (2022) 36:108127. doi: 10.1016/j.jdiacomp.2022.108127, PMID: 35067449

[54] Kalantar-Zadeh K Rhee CM Chou J Ahmadi SF Park J Chen JL. The obesity paradox in kidney disease: how to reconcile it with obesity management. Kidney Int Rep. (2017) 2:271–81. doi: 10.1016/j.ekir.2017.01.009, PMID: 28439569 PMC5399774

[55] Johansen KL Lee C. Body composition in chronic kidney disease. Curr Opin Nephrol Hypertens. (2015) 24:1–75. doi: 10.1097/MNH.0000000000000120, PMID: 25887900 PMC4778545

[56] Kataoka H Nitta K Hoshino J. Visceral fat and attribute-based medicine in chronic kidney disease. Front Endocrinol (Lausanne). (2023) 14:1097596. doi: 10.3389/fendo.2023.1097596, PMID: 36843595 PMC9947142

[57] Purnamasari D Tetrasiwi EN Kartiko GJ Astrella C Husam K Laksmi PW. Sarcopenia and chronic complications of type 2 diabetes mellitus. Rev Diabet Stud. (2022) 18:157–65. doi: 10.1900/RDS.2022.18.157, PMID: 36309772 PMC9652710

[58] Braga TT Forni MF Correa-Costa M Ramos RN Barbuto JA Branco P. Soluble uric acid activates the nlrp3 inflammasome. Sci Rep. (2017) 7:39884. doi: 10.1038/srep39884, PMID: 28084303 PMC5233987

[59] Govaere O Petersen SK Martinez-Lopez N Wouters J Van Haele M Mancina RM. Macrophage scavenger receptor 1 mediates lipid-induced inflammation in non-alcoholic fatty liver disease. J Hepatol. (2022) 76:1001–12. doi: 10.1016/j.jhep.2021.12.012, PMID: 34942286 PMC7619241

[60] Lau WB Ohashi K Wang Y Ogawa H Murohara T Ma XL. Role of adipokines in cardiovascular disease. Circ J. (2017) 81:920–8. doi: 10.1253/circj.CJ-17-0458, PMID: 28603178

[61] Johnson RJ Sanchez LL Lanaspa MA Piani F Borghi C. Uric acid and chronic kidney disease: still more to do. Kidney Int Rep. (2023) 8:229–39. doi: 10.1016/j.ekir.2022.11.016, PMID: 36815099 PMC9939362

[62] Leyane TS Jere SW Houreld NN. Oxidative stress in ageing and chronic degenerative pathologies: molecular mechanisms involved in counteracting oxidative stress and chronic inflammation. Int J Mol Sci. (2022) 23:23. doi: 10.3390/ijms23137273, PMID: 35806275 PMC9266760

[63] Fu YC Xu ZL Zhao MY Xu K. The association between smoking and renal function in people over 20 years old. Front Med (Lausanne). (2022) 9:870278. doi: 10.3389/fmed.2022.870278, PMID: 35721101 PMC9205397

[64] Yilmaz M Kayancicek H. A new inflammatory marker: elevated monocyte to HDL cholesterol ratio associated with smoking. J Clin Med. (2018) 7:7. doi: 10.3390/jcm7040076, PMID: 29642607 PMC5920450

[65] Wang L Xu Y Zhang X Ding J Jin J Zong J. The predictive value of SII combined with UHR for contrast-induced acute kidney injury in patients with acute myocardial infarction after percutaneous coronary intervention. J Inflamm Res. (2024) 17:7005–16. doi: 10.2147/JIR.S482977, PMID: 39372595 PMC11456302

