# Supplementary material for: Association between uric acid to high-density lipoprotein cholesterol ratio and chronic kidney disease in Chinese patients with type 2 diabetes mellitus: a cross-sectional study
Source: Front Nutr. 2025 Apr 14;12:1582495. doi: 10.3389/fnut.2025.1582495 (PMC12034545; doi:10.3389/fnut.2025.1582495)
Supplement: Supplementary file 1 [file Data_Sheet_1.ZIP › Supplementary file.doc]

**Supplementary:**

**Table S1. Assessment of multicollinearity among independent variables (*n*=1,756)**

| **Variables** | **GVIF** | **Df** | **GVIF^1/2Df^** |
| --- | --- | --- | --- |
| UHR | 1.469748 | 1 | 1.212332 |
| Sex | 1.684825 | 1 | 1.298008 |
| Age | 1.260351 | 1 | 1.122654 |
| Education attainment | 1.120115 | 2 | 1.028764 |
| BMI | 1.220711 | 1 | 1.104858 |
| Alcohol drinking | 1.195117 | 1 | 1.093214 |
| Smoking | 1.495416 | 1 | 1.222872 |
| Elevated TC | 1.623772 | 1 | 1.274273 |
| Elevated TG | 1.369394 | 1 | 1.170211 |
| Elevated LDL-C | 1.547796 | 1 | 1.244105 |
| Hypertension | 1.162879 | 1 | 1.078369 |
| Diabetes duration | 1.072591 | 1 | 1.035660 |
| Elevated HbA1c | 1.365871 | 1 | 1.168705 |
| Elevated FPG | 1.347132 | 1 | 1.160660 |

Abbreviations: UHR, uric acid to high-density lipoprotein cholesterol ratio; BMI, body mass index; TC, total cholesterol; TG, triglycerides; LDL-C, low-density lipoprotein cholesterol; HbA1c, hemoglobin A1c; FPG, fasting plasma glucose;

**Table S2. Covariates before and after PSM**

| **Variables** | **Level** | **Before Matching** | | | **After Matching** | | |
| --- | --- | --- | --- | --- | --- | --- | --- |
|  |  | **Group without CKD** | **Group CKD** | **SMD** | **Group without CKD** | **Group CKD** | **SMD** |
| n |  | 1,271 | 485 |  | 485 | 485 |  |
| BMI (mean (SD)) |  | 24.51 (3.35) | 25.43 (3.56) | 0.258 | 25.33 (3.53) | 25.43 (3.56) | 0.029 |
| Age (mean (SD)) |  | 56.52 (9.92) | 59.09 (10.54) | 0.244 | 58.81 (8.91) | 59.09 (10.54) | 0.026 |
| Sex (%) | Female | 639 (50.3) | 241 (49.7) | -0.012 | 250 (51.5) | 241 (49.7) | -0.037 |
|  | Male | 632 (49.7) | 244 (50.3) | 0.012 | 235 (48.5) | 244 (50.3) | 0.037 |
| Elevated HbA1c (%) | No | 691 (54.4) | 206 (42.5) | -0.241 | 212 (43.7) | 206 (42.5) | -0.025 |
|  | Yes | 580 (45.6) | 279 (57.5) | 0.241 | 273 (56.3) | 279 (57.5) | 0.025 |
| Hypertension (%) | No | 557 (43.8) | 100 (20.6) | -0.574 | 108 (22.3) | 100 (20.6) | -0.041 |
|  | Yes | 714 (56.2) | 385 (79.4) | 0.574 | 377 (77.7) | 385 (79.4) | 0.041 |
| Elevated TC (%) | No | 1203 (94.6) | 433 (89.3) | -0.174 | 437 (90.1) | 433 (89.3) | -0.027 |
|  | Yes | 68 (5.4) | 52 (10.7) | 0.174 | 48 (9.9) | 52 (10.7) | 0.027 |
| Elevated TG (%) | No | 949 (74.7) | 303 (62.5) | -0.252 | 306 (63.1) | 303 (62.5) | -0.013 |
|  | Yes | 322 (25.3) | 182 (37.5) | 0.252 | 179 (36.9) | 182 (37.5) | 0.013 |
| Elevated LDL-C (%) | No | 1201 (94.5) | 449 (92.6) | -0.073 | 450 (92.8) | 449 (92.6) | -0.008 |
|  | Yes | 70 (5.5) | 36 (7.4) | 0.073 | 35 (7.2) | 36 (7.4) | 0.008 |
| Elevated FPG (%) | No | 595 (46.8) | 172 (35.5) | -0.237 | 171 (35.3) | 172 (35.5) | 0.004 |
|  | Yes | 676 (53.2) | 313 (64.5) | 0.237 | 314 (64.7) | 313 (64.5) | -0.004 |
| Education attainment (%) | Secondary education or below | 1130 (88.9) | 411 (84.7) | -0.116 | 416 (85.8) | 411 (84.7) | -0.029 |
|  | Senior high school | 108 (8.5) | 63 (13.0) | 0.134 | 59 (12.2) | 63 (13.0) | 0.025 |
|  | College education or above | 33 (2.6) | 11 (2.3) | -0.022 | 10 (2.1) | 11 (2.3) | 0.014 |
| Smoking (%) | No | 953 (75.0) | 367 (75.7) | 0.016 | 373 (76.9) | 367 (75.7) | -0.029 |
|  | Yes | 318 (25.0) | 118 (24.3) | -0.016 | 112 (23.1) | 118 (24.3) | 0.029 |
| Alcohol drinking (%) | No | 785 (61.8) | 325 (67.0) | 0.112 | 325 (67.0) | 325 (67.0) | 0.000 |
|  | Yes | 486 (38.2) | 160 (33.0) | -0.112 | 160 (33.0) | 160 (33.0) | 0.000 |
| Diabetes duration (%), years | <=10 | 999 (78.6) | 328 (67.6) | -0.234 | 337 (69.5) | 328 (67.6) | -0.040 |
|  | >10 | 272 (21.4) | 157 (32.4) | 0.234 | 148 (30.5) | 157 (32.4) | 0.040 |

Abbreviations: CKD, chronic kidney disease; PSM, propensity score matching; SMD, standardized mean difference; BMI, body mass index; TC, total cholesterol; TG, triglycerides; LDL-C, low-density lipoprotein cholesterol; HbA1c, hemoglobin A1c; FPG, fasting plasma glucose.

**Table S3. Multivariable regression analysis of UHR tertiles and its clusters in relation to CKD after PSM (*n*=970)**

|  | Model1 | | Model2 | | Model3 | |
| --- | --- | --- | --- | --- | --- | --- |
|  | OR(95%CI) | *p* | OR(95%CI) | *p* | OR(95%CI) | *p* |
| UHR per SD | 1.32(1.15-1.51) | <0.001 | 1.30(1.13-1.49) | <0.001 | 1.40(1.19-1.64) | <0.001 |
| UHR tertile |  |  |  |  |  |  |
| T1 | 1.00(ref) |  | 1.00(ref) |  | 1.00(ref) |  |
| T2 | 1.03(0.76-1.40) | 0.848 | 1.02(0.75-1.40) | 0.886 | 1.10(0.79-1.52) | 0.570 |
| T3 | 1.72(1.26-2.35) | <0.001 | 1.64(1.18-2.27) | 0.003 | 1.86(1.28-2.70) | 0.001 |
| P for trend | 1.05(1.02-1.08) | <0.001 | 1.05(1.02-1.08) | <0.001 | 1.06(1.02-1.09) | <0.001 |

Abbreviations: UHR, uric acid to high-density lipoprotein cholesterol ratio; CKD, chronic kidney disease; PSM, propensity score matching; OR, odds ratio; CI, confidence interval; SD, standard deviation; T1, first tertile; T2, second tertile; T3, third tertile; ref, reference.

Model 1: unadjusted analysis (no covariates included); Model 2: adjusted for age, sex;

Model 3: fully adjusted model, incorporating age, sex, education, body mass index, unfavorable lipid profile (total cholesterol, triglyceride, low-density lipoprotein cholesterol) and glycemic parameters (hemoglobin A1c, fasting plasma glucose), hypertension, smoking, alcohol drinking, and diabetes duration.

Figure S1. K-means clustering method for UHR levels.

**Table S4. Multivariable regression analysis of UHR clusters in relation to CKD (*n*=1,756)**

| UHR cluster  (range) | Model 1 | | Model 2 | | Model 3 | |
| --- | --- | --- | --- | --- | --- | --- |
|  | OR(95%CI) | *p* | OR(95%CI) | *p* | OR(95%CI) | *p* |
| Cluster 1 (2.09-10.34) | 1.00(ref) |  | 1.00(ref) |  | 1.00(ref) |  |
| Cluster 2 (10.34-16.28) | 1.22(0.95-1.58) | 0.114 | 1.27(0.98-1.64) | 0.068 | 1.17(0.89-1.55) | 0.261 |
| Cluster 3 (16.30-25.68) | 2.21(1.66-2.93) | <0.001 | 2.35(1.74-3.18) | <0.001 | 1.93(1.37-2.73) | <0.001 |
| Cluster 4(25.82-55.50) | 4.44(2.64-7.49) | <0.001 | 4.83(2.83-8.24) | <0.001 | 3.35(1.84-6.11) | <0.001 |
| P for trend | 1.07(1.05-1.09) | <0.001 | 1.08(1.06-1.10) | <0.001 | 1.06(1.03-1.08) | <0.001 |

Abbreviations: UHR, uric acid to high-density lipoprotein cholesterol ratio; CKD, chronic kidney disease; OR, odds ratio; CI, confidence interval; ref, reference.

Model 1: unadjusted analysis (no covariates included); Model 2: adjusted for age, sex;

Model 3: fully adjusted model, incorporating age, sex, education, body mass index, unfavorable lipid profile (total cholesterol, triglyceride, low-density lipoprotein cholesterol) and glycemic parameters (hemoglobin A1c, fasting plasma glucose), hypertension, smoking, alcohol drinking, and diabetes duration.

**Table S4. Threshold efect analysis of UHR on CKD in lower BMI group (BMI < 24kg/m^2^) (*n*=734)**

|  | OR (95% CI)* | p |
| --- | --- | --- |
| **Fitting by standard Logistic regression model** | 1.09 (1.05, 1.13) | <0.001 |
| **Fitting by piecewise Logistic regression model (break-point = 5.85)** |  |  |
| UHR < 5.85 | 0.65 (0.44, 0.95) | 0.028 |
| UHR≥ 5.85 | 1.11 (1.06, 1.16) | <0.001 |
| **Log likelihood ratio** |  | **0.012** |

Abbreviations: UHR, uric acid to high-density lipoprotein cholesterol ratio; CKD, chronic kidney disease; BMI, body mass index; OR, odds ratio; CI, confidence interval; ref, reference.

*Adjusted for age, sex, education, unfavorable lipid profile (total cholesterol, triglyceride, low-density lipoprotein cholesterol) and glycemic parameters (hemoglobin A1c, fasting plasma glucose), hypertension, smoking, alcohol drinking, and diabetes duration.
